# Relating Wildlife Camera Trap Data to Tick Abundance: Testing the Relationship in Different Habitats

**DOI:** 10.3390/ani14182749

**Published:** 2024-09-23

**Authors:** Rachele Vada, Stefania Zanet, Flavia Occhibove, Enrica Fantini, Pablo Palencia, Ezio Ferroglio

**Affiliations:** Department of Veterinary Sciences, University of Turin, 10095 Grugliasco, Italy

**Keywords:** camera traps, tick abundance, tick–host association

## Abstract

**Simple Summary:**

This study addresses the growing risk of tick bites, which is becoming more prevalent due to changes in landscape, leading to an increase in wildlife that supports tick populations, raising the risk of disease transmission to humans and animals. To better understand this, we explored the ecology of ticks by examining the relationship between wildlife presence and tick abundance in two regions: an alpine hunting district and a natural park in the Apennines. We used camera traps to monitor wildlife and conducted tick sampling nearby. Additionally, we considered factors like altitude, vegetation, and climate. Our findings highlight the significant role of altitude and wildlife presence—both influenced by habitat and land management—in the global abundance of ticks in the environment. In particular, the species most impacting tick presence was roe deer. These insights could be valuable for managing natural environments, helping to reduce the risk of tick-borne diseases, and they show the utility of camera trap data, which are gathered with great detail and little disturbance of wildlife.

**Abstract:**

The increase in acarological risk of tick bites is significantly driven by profound changes in landscape, which alter the density and distribution of wildlife that support tick populations. As a result of habitat shifts and land abandonment, which create environments conducive to tick proliferation, the risk of disease transmission to humans and animals is increasing. In this context, it is important to explore tick ecology by applying a comprehensive methodology. In this study, we examined the relationship between wildlife temporal occupancy and tick abundance in two distinct regions: an alpine hunting district and a natural park in the Apennines. For each sampling point, we calculated wildlife temporal occupancy from camera trap pictures and estimated ticks’ abundance from dragging transects in the area immediately surrounding camera traps. In modelling the relationship between those two variables, we included abiotic factors such as saturation deficit, normalized difference vegetation index, and altitude. Results show the importance of altitude and wildlife temporal occupancy (itself related to different habitat and land management characteristics) on the ecology of questing ticks. If employed in management decisions for natural environments, such information is useful to modulate the acarological risk and thus the risk of tick-borne pathogens’ transmission.

## 1. Introduction

The recent altitudinal and latitudinal expansion of tick populations has become a significant concern due to the zoonotic pathogens they carry, which are classified as emerging infectious diseases [1,2]. By influencing different aspects of tick biology (survival, development, reproduction, and dispersal), abiotic factors, habitat (in a broad sense, including human interventions), and the presence of wild vertebrate hosts are responsible for this range expansion of ticks [3].

The altitudinal factor allows us to study ticks’ abundance at the extremes of their distribution and therefore draw conclusions on how climate change will impact those vectors. For instance, higher altitudes appear to constrain tick eggs hatching and molting to further stages [4], even independently from the host presence [5].

In addition, habitat or landscape modification, through its impact on tick hosts’ presence and abundance, may determine ticks’ presence and expansion, as well as impact their abundance [3]. Scientific work and anecdotical considerations (collected by Medlock and colleagues [3]) show how the tick population has increased across Europe, probably linked to the impact of human activities on the environment and habitat modification. In addition to this, wildlife management can be, in some cases, a strategy to reduce the acarological risk for people. While some studies have shown that deer removal (either by culling or fencing) can reduce the number of immature stages of ticks in the environment [4,5,6], this relationship may be weak in some contexts [7] or with temporal limitations [8].

With an increasing trend in the last few years, camera traps have been claimed by the scientific community to be the most efficient tool to monitor wildlife in terms of cost-effectiveness and reliability of results [9]. They have been integrated into studies addressing tick ecology worldwide, thanks to the high quality of wildlife data that they can gather [10,11,12,13,14], sometimes also integrating small mammal trapping [15,16]. Hofmeester and colleagues [17] implemented camera traps to relate wildlife passage rates to tick abundance, highlighting a positive correlation and introducing camera trap data to the study of the relationship between the densities of questing ticks and the availability of different vertebrate species.

In this scenario, it is important to apply holistic approaches to study the ecology of ticks and, consequentially, tick-borne diseases. In the present work, we aim to examine the relationship between ticks’ abundance and host presence, by implementing the high-quality data obtained from camera traps to estimate wild ungulates’ and mesocarnivores’ temporal occupancy (TO). Findings may be of use as tools to decrease the risk of tick biting in decision-making processes for habitat and wildlife management.

## 2. Materials and Methods

### 2.1. Study Areas

No ethical approval was necessary for this study, as it did not involve any direct manipulation of or intervention with wild animals.

Two study areas in the same geographical region (northwest Italy) were selected: Val Maira Hunting District Cuneo 3 (CACN3—Italy) and Capanne di Marcarolo Natural Park (Aree Protette Appennino Piemontese, Italy). Val Maira is an alpine Hunting District comprising 56,700 Ha, featuring an open valley with a mean altitude of 1650 m a.s.l. (considered in our study area from 500 m a.s.l. to 2500 m a.s.l.). The annual precipitation for 2020 was 788.2 mm, and the mean temperature was 10.19 °C. The valley registers a population of 11.500 inhabitants (20.28 inh/km^2^) and several agropastoral activities. Capanne di Marcarolo is a natural park comprising 8216 Ha in the Appennines, with a mean altitude of 754 m a.s.l. (from 300 m a.s.l. to 1000 m a.s.l.). The annual precipitation for 2020 was 1612.4 mm, and the mean temperature was 10.92 °C. Around 30 people reside in the park the whole year long and several agropastoral activities are present. Wild ungulate density for those areas was studied in the framework of the European Wildlife Observatory [18] and estimated contextually to the sampling for the present work. In Val Maira, there is a higher density of wild boar (10.09 ± 3.37 in/km^2^) and roe deer (15.9 ± 4.38 in/km^2^), with a lower density of red deer (0.79 ± 0.77 in/km^2^). Density for roe deer in Capanne di Marcarolo is 6.35 ± 1.88 in/km^2^, and for wild boar, density is 0.27 ± 0.11 in/km^2^, while no red deer was recorded in the park (Palencia et al., 2023a). Other species present in both areas are red fox (*Vulpes vulpes*), badger (*Meles meles*), European pine marten (*Martes martes*), and beech marten (*Martes foina*).

### 2.2. Sampling Design

**Figure 1:**
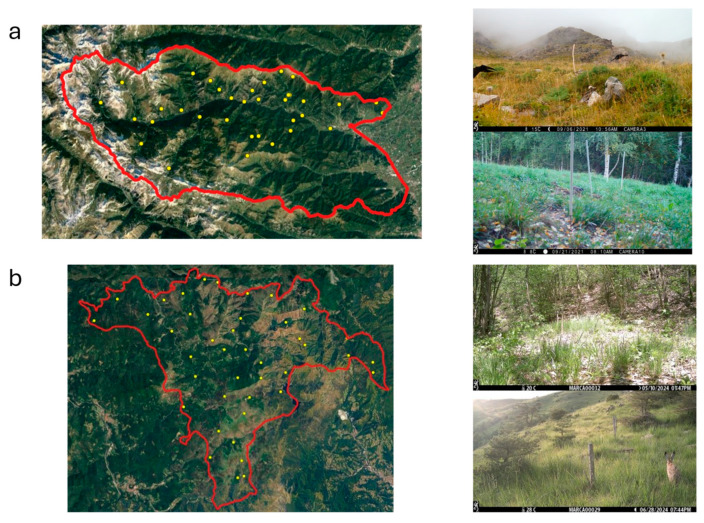
Borders of Val Maira (**a**) and Capanne di Marcarolo (**b**) study areas, with sampling points’ distribution. Pictures on the right show the type of habitat sampled.

To homogenously cover the study area and its macrohabitats with an adequate number of camera traps (we used Browing FORCE EDGE—Model BTC-7E, or Browing DARK OPS APEX—Model BTC-6HD-APX), we selected a total of 38 sampling points in Val Maira and 41 in Capanne di Marcarolo (Figure 1). Sampling points were selected in closed (forest) and open (meadows) habitats, proportionally to their extension in each park. Cameras were deployed facing north, 50 cm above the ground, with the sensor angled parallel to the slope. Cameras were set to be operative all day, to record a burst of eight consecutive pictures (rapid fire setting) at each activation, with the minimum time laps (0.22 s) between consecutive activations. Nocturnal pictures were illuminated with infrared flash (low glow). Neither baits nor attractants were used. The date and time of each capture were automatically stamped onto each picture.

**Figure 2:**
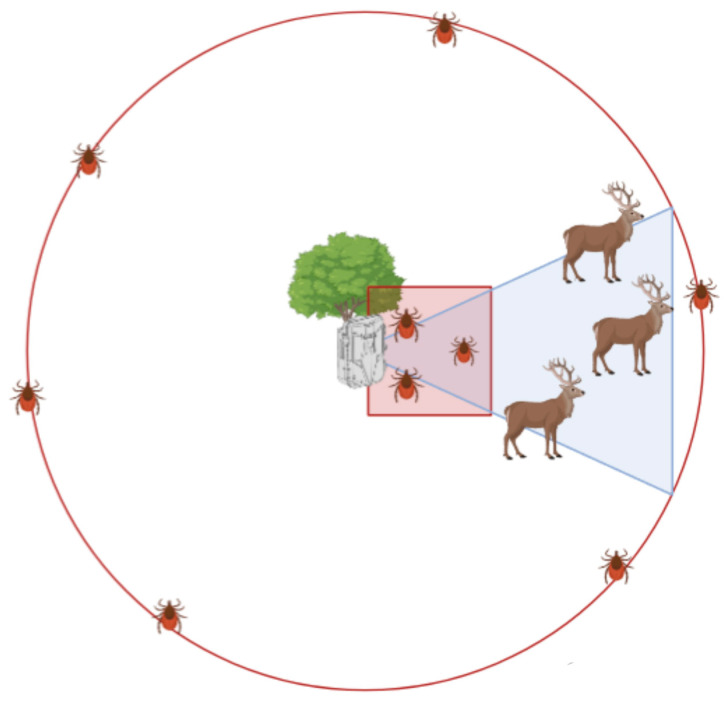
Graphical representation of the sampling design, including tick-dragging transects referred to the camera trap deployment and field of view. Animals that are in the field of view are recorded via the camera trap, while a 100 m^2^ (10 × 10 m) square dragging transect is performed in front of it, and a dragging transect in a circle with a 26 m radius is performed around the camera trap.

Questing ticks were collected with two tick-dragging transects, in a 10 m^2^ square and a 26 m radius circle, respectively, in front and around the CT (Figure 2), and a data logger (Temperature and Humidity Data Logger RS PRO, USB) was set to register hourly the temperature and humidity 15 cm above the ground. We performed a first tick-dragging transect (T_t_) at the deployment of the CT, to clean the area from questing ticks, and a second transect (T_1_) after one month, at the removal of the CT. This duration was chosen to strike a balance between collecting enough wildlife data for all species (including wild boar, which were less abundant in Capanne di Marcarolo) and obtaining tick abundance data with sufficient resolution to be correlated with wildlife TO. Due to the need to avoid hunting and other activities in the study areas, in Val Maira, this study was performed from the end of August 2021 to the beginning of October 2021, and camera traps were rotated to cover all sampling points, while in Capanne di Marcarolo it was performed in July 2022.

### 2.3. Data Preparation

For statistical purposes, we considered an observation as the sum of data collected at each sampling point at T1. Therefore, each observation was characterized by the following: (a) tick abundance from both transects at T1, (b) wildlife temporal occupancy (TO), (c) mean normalized difference vegetation index (NDVI), (d) maximum saturation deficit (maxSD), and (e) altitude. Data were prepared as follows:

(a) Tick abundance. Ticks collected from dragging were stored in ethanol 70% and later identified with morphological keys [19,20,21]. Ticks were identified with a Nikon SMZ1500 stereomicroscope (Nikon Corporation, Tokyo, Japan) and Zeiss Axiolab 5 microscope (Carl Zeiss AG, Oberkochen, Germany). Only ticks collected at T1 were considered in the analysis, and abundance was calculated with the sampling point referring to the total area sampled via the two dragging transects;
(b) Wildlife TO. We focused on species that had good detectability in camera traps, thus extracting TO for wild boar, red deer, roe deer, red fox, badger, pine marten, and beech marten. Camera trap pictures were analyzed to extract, for each individual, the following information: sampling point, date of passage, and time spent in front of the camera (calculated as the number of seconds between the first picture and the last). As the aim of this analysis was to calculate the species TO, no individual recognition was required and, for simplicity, an individual exiting the field of view and entering it again was considered as a new individual, so that only the actual time spent in front of the camera trap was considered. In the case that more than one individual appeared in the picture, the time of each animal was calculated separately and eventually summed. As species were unevenly represented in the two study areas, we merged seconds per sampling point for mesocarnivores, and we decided to discard the red deer for its reduced presence and being localized in one study area only, thus implementing in the analysis the TO for roe deer, wild boar, and mesocarnivores;
(c) Mean NDVI. These data were retrieved with the R [22] package MODIStsp [23] at a resolution of 250 m and 16-day intervals. Dates were matched with sampling dates and point coordinates, and the mean was calculated;
(d) MaxSD. We registered, for each sampling point, the daily mean temperature and humidity, and calculated the daily mean SD (as in [24] per sampling point, from which we derived the maxSD per sampling point in the whole period. Mean and minimum SD were considered but ultimately discarded, based on the high collinearity with max SD and considering that the max SD was the most limiting factor to tick activity [25];
(e) Altitude. Altitude was registered with a GPS device (GPSmap 60CSx, Garmin Ltd., Olathe, KS, USA) for each sampling point.

We also retrieved CORINE (coordination of information on the environment) land cover (CLC) data from Geoportale Piemonte (https://www.geoportale.piemonte.it/cms/progetti/land-cover-piemonte, accessed on 21 September 2023). To limit variability, we grouped the CLC data (from the LCP level) into three classes: evergreen forests, deciduous forests, and human activities patches (pastures).

### 2.4. Statistical Analysis

We modelled the impact of six explanatory variables (max SD, mean NDVI, TO for wild boar, roe deer, and mesocarnivores, and altitude) on tick abundance, adding the site as a further explanatory variable. We cleaned the database of outliers and points where none of the species of our interest was recorded. We also checked data for normality and applied to each variable a normalization transformation when needed, with the bestNormalize package [26] in R studio. Relationships among explanatory and response variables were modelled using GAMMs (General Additive Mixed Models [27]). GAMMs extend generalized linear models (GLMs/GLMMs) by using smooth functions to define nonlinear relationships between the response and explanatory variables, and by combining predictor variables additively, which makes them particularly suitable for ecological study [27,28]. We tested the performance of the variables as linear predictors and smooth terms, while, however, expecting a linear relationship with maxSD, altitude, or mean NDVI, and a nonlinear relationship with wildlife TO. We also tested the effects of the interaction between different variables. We tested spatial random effects for the sampling point. Moreover, we explored different variance weights, including altitude, site, and interactions between them. To consider sampling area differences, the site was implemented in interaction with other explanatory variables. Model selection was based on three parameters: (i) the statistically significant (ANOVA test) lowest AIC (Akaike information criterion), (ii) a satisfactory R2 (considering the complexity of the biological phenomena observed), and (iii) good performance of residuals analysis and smooth graphs. Analysis was performed in R studio with the function gam of the mgcv package [27].

After analyzing the impact of wildlife TO on tick abundance, we also tested differences in wildlife TO in relation to CLC classes and altitude with a Kruskal–Wallis test [29] due to the non-normality of data.

## 3. Results

### 3.1. Tick Transects and Camera Trap Pictures

We registered a total of 32 observations in Val Maira and 37 in Capanne di Marcarolo. The majority of ticks were in larval stages. The most abundant genera were *Ixodes* (*I. ricinus*) and *Haemaphysalis* (*H. punctata* and *H. concinna*). *Ixodes* spp. comprised 96% of the ticks collected in Val Maira and 46% in Capanne di Marcarolo, while *Haemaphysalis* spp. made up 4% of collected ticks in Val Maira and 54% in Capanne di Marcarolo (Table 1). The difference in tick abundance among the two areas was not statistically significant (Kruskal–Wallis, *p* < 0.05). However, Val Maira registered a lower average abundance (1.45 ticks/m^2^) than Capanne di Marcarolo (3.37 ticks/m^2^).

**Figure 3:**
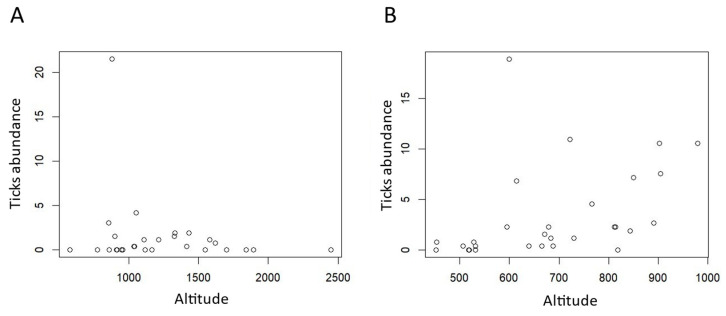
Dotplot of tick abundance in relation to altitude. (**A**) Val Maira and (**B**) Capanne di Marcarolo.

Tick abundance varied differently in the two areas according to altitude. In Val Maira, we registered a peak between 1000 m a.s.l. and 1700 m a.s.l. (Figure 3A), while in Capanne di Marcarolo the abundance increased more homogeneously with altitude (Figure 3B).

The highest temporal occupancy was registered for roe deer, followed by wild boar, and we recorded a low presence of red deer (or absence, as in the case of Capanne di Marcarolo), although with great differences among the two study areas (Table 2). Mesocarnivores were registered in most sampling points.

### 3.2. Temporal Occupancy Model

**Figure 4:**
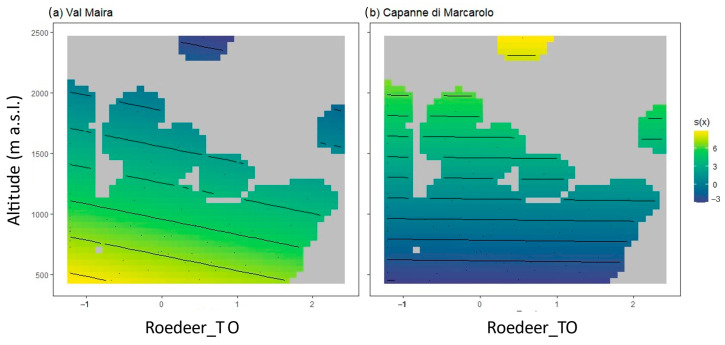
2D smooth effect plots. Smooth effects on tick abundance are presented for roe deer TO and altitude at Val Maira (**a**) and Capanne di Marcarolo (**b**). Temporal occupancy ranges from negative to positive values due to transformations to normalize data. In b, data above 980 m a.s.l. are an interpolation of the model, as no information was recorded in the park.

Following best model selection through R^2^, AIC, and residual analysis (Tables S1 and S2, Figure S1), tick abundance was best predicted with mesocarnivores TO, maxSD, meanNDVI, and altitude as linear predictors; however, only the latter showed a statistically significant (negative) impact on tick abundance. Wildlife TO for roe deer and wild boar was implemented in the model as a nonlinear predictor, interacting with altitude separately by the site. Altitude showed a significant detractive impact, while NDVI had an additive impact, although not statistically significant. For smooth terms, the only significance was recorded for roe deer TO. In Val Maira, we recorded an additive effect at lower altitudes and lower roe deer TO, and it progressively decreased until it became a detractive effect (Figure 4a). In Capanne di Marcarolo, the roe deer effect is less marked (Figure 4b). No data were recorded in Capanne di Marcarolo above 980 m a.s.l., and the effect is detractive at low altitudes.

### 3.3. Variations of TO

**Figure 5:**
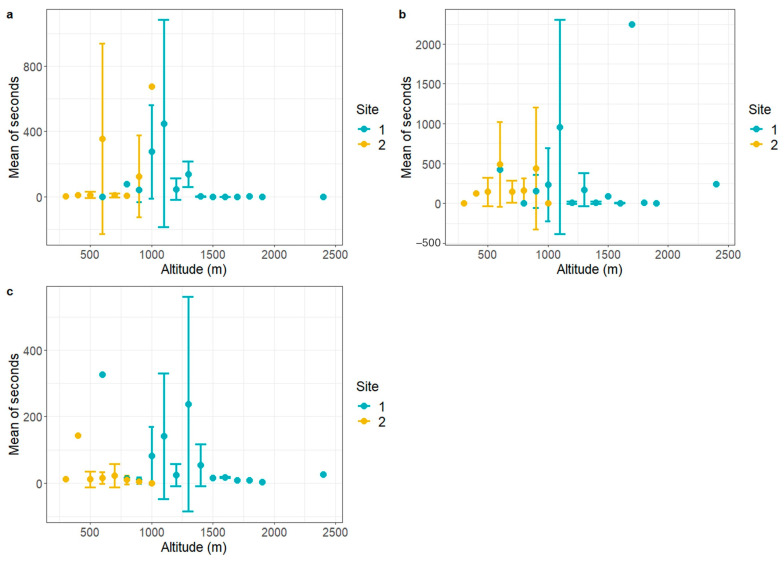
Mean TO according to altitude. Mean TO for 100 m altitude gradient was calculated with relative error bars for wild boar (**a**), roe deer (**b**), and mesocarnivores (**c**), separated for Val Maira (Site 1) and Capanne di Marcarolo (Site 2).

The only significant difference among wildlife TOs in the CLC classes was for wild boar, which seem to be spending more time in deciduous forests in Val Maira. Regarding the fluctuation of TO according to altitude, the range from 1000 m a.s.l. to 1500 m a.s.l. records the highest presence for all species (Figure 5). However, roe deer seem to be particularly active both at lower and, though with less intensity, higher altitudes.

## 4. Discussion

In this study, we modelled the impact of wildlife TO on tick abundance in two different study areas: an alpine hunting district, where the altitude gradient is particularly broad, impacting the habitat and wildlife population, and an Apennine natural park, with lower biodiversity for ungulate species, a drier climate. We note that the impact of wild species on tick abundance depends on whether they are a preferential host or not, but we highlight a much more important role for abiotic factors, such as altitude.

The two most abundant genera are *Ixodes* and *Haemaphysalis*. In Val Maira, there is a neat predominance of *Ixodes ricinus*, a result which is anyways consistent with the literature findings [30]. However, the trend inverts in Capanne di Marcarolo, where *H. punctata* becomes the main species, almost matching the number of *I. ricinus*. This supports *Haemaphysalis* spp. being reported mainly in the Mediterranean area, as well as its preference for humid and well-vegetated areas [30]; in Capanne di Marcarolo, most ticks were found in deciduous forests with shrubs or dead leaves to keep humidity, and little findings were reported in other open habitats, which, in a Mediterranean habitat, are too dry for tick activity.

GAMMs are particularly useful to model the nonlinear relationship that often occurs in non-biological environments. This approach, as implemented, for example, by Elias and colleagues [28], offers a significant benefit over Generalized Linear Mixed Models (GLMMs), which are limited to modelling linear relationships, or simpler statistical correlations that do not account for all variables simultaneously—an essential consideration when investigating complex phenomena. In our study, we captured a snapshot of the relationship between wildlife presence and tick abundance by constructing a global model that included all tick stages without differentiation. However, for more detailed investigations, future studies should consider incorporating hatching and molting times at different altitudes and environmental conditions, to more precisely correlate wildlife presence with different tick developmental stages. Furthermore, expanding the study design to include additional host species, such as small mammals crucial to tick maintenance in the environment, could enhance the breadth of the research by targeting a wider host spectrum. Regarding wild boar, we did not record any statistically significant effect in the model, which may be in accordance with the scarce role of this species in supporting *I. ricinus* population, not being a preferential host [31,32,33]. Wild boar presence in Capanne di Marcarolo is rather limited [34], and the few individuals left in the park were not sufficient to play a role in impacting tick abundance anyways. Similarly, mesocarnivores also do not show any statistically significant impact, probably due to their scarce TO in the sampled areas compared to roe deer.

According to the model, ticks’ abundance decreased with increasing altitude, where the environment becomes less favorable for them [35]. When looking at the data, we can spot, instead, a more bell-shaped trend, with an increase up to 1000 m a.s.l. and a decrease after 1700 m a.s.l., registering the highest abundance where temperature and humidity combine with moderate altitude. Indeed several studies on *I. ricinus* in mountain areas of different regions have shown how altitude has a detractive impact on ticks presence [4,5,36,37,38,39], also influencing developmental/eggs’ hatching time and success [4]. Of course, such behavior is particularly interesting under the scope of climate change threats, as increasing temperatures are creating favorable habitats for ticks at higher altitudes, shifting uphill their limit of distribution [3,38,40]. The impact on ticks’ presence and activity of parameters such as exposition of the slope [41] and solar radiation [42] has previously been proved, which we also tested in the model, but were ultimately not added due to poor performance of the outputs and statistical similarity with altitude effects.

Habitat characteristics, especially vegetation presence on the ground and tree/shrub cover (higher NDVI), have shown, together with altitude, a positive impact on tick abundance. The lack of statistical significance can be related to the scale of the NDVI (250 m) being broader than the actual sampled area (10 m^2^ and 26 m radius circle) and therefore, in some cases, not representing the actual vegetation cover for the specific sampled area. Indeed, such a relationship (NDVI positively correlating with tick abundance) was already highlighted by other works in Europe [43,44,45,46], as vegetation can maintain a better balance of temperature and humidity for the survival and activity of ticks. Also, larger mammals tend to spend more time resting or looking for food in covered areas (wild boar for instance were recorded significantly more in deciduous forests), which could relate to the higher abundance of ticks.

Altitude seems to have the highest impact on tick abundance, with a moderate effect of the interaction with roe deer. In Capanne di Marcarolo, the effect at higher altitudes is indeed just an interpolation of the model. Below 1000 m a.s.l. a detractive impact is still reported, despite not being related to the increase in temporal occupancy: the effect of roe deer in Capanne di Marcarolo is overall quite negligible. In Val Maira, roe deer temporal occupancy increases ticks’ abundance at low altitudes, with a progressively less-strong additive impact with the increase in temporal occupancy. At higher altitudes, this effect converts into being progressively more detractive. Apparently, with the principal limiting element for ticks’ activity being abiotic factors, the summatory presence of the host may remove ticks from the environment: the more time deer species spend in front of a camera trap, the more ticks tend to jump on the host, especially at higher altitudes, while at a lower altitude, even brief roe deer presence still adds environmental ticks. Such a result does not surprise if we consider that those species are preferential hosts for *I. ricinus* and *H. punctata*. Since deer species are a preferential host for the main tick species collected in this study, and the sampling periods in both study areas exhibit a decreasing phase in ticks’ activities, it is possible that the lower host density, the broader range of different habitats and altitudes, and the scattered wildlife presence in a wider sampling area all contribute to this different result.

A significant difference in terms of tick abundance and model outputs can be spotted between the two study areas. Despite their similarities, differences in wildlife densities and habitat types may still have significant impacts on the number of questing ticks in the environment and the tick–host relationship.

## 5. Conclusions

In the present work, we highlight how environmental characteristics (habitat macro type and its consequences on climate and vegetation, altitude, land use, and management) that ultimately affect wildlife density and distribution (constraining its movements or focusing its presence in particular areas) may affect the relationship between wildlife TO and the abundance of questing ticks. Since habitat and wildlife management is the most useful tool to impact the risk of tick bites for people in the natural environment, our results highlight the importance of contextualizing the ecology of the ticks in relationship with their vertebrate hosts to the specificities of the habitats for decision-making on habitat intervention.

## Figures and Tables

**Table 1:** Total number of ticks collected with dragging, per developmental stage and species.

	Val Maira	Capanne di Marcarolo
Larvae	143	735
Nymphs	49	21
Adults	6	3
*Ixodes ricinus*	190	350
*Haemaphysalis punctata*	7	406
*Haemaphysalis concinna*	0	3
*Dermacentor reticulatus*	1	0

**Table 2:** Total number of seconds registered via camera traps by species and sampling area. In parenthesis is the number of sampling points where at least one individual of the species was recorded. As camera trap pictures often do not enable distinguishing between stone and pine marten, mesocarnivores seconds were aggregated.

	Val Maira	Capanne di Marcarolo
Mesocarnivores	2644 (26/38)	511 (20/41)
Red deer	818 (8/38)	Not present
Roe deer	7607 (20/38)	6361 (26/41)
Wild boar	3021 (16/38)	2432 (18/41)

## Data Availability

Data will be made available upon reasonable request to the authors.

## Supplementary Materials

The following supporting information can be downloaded at: https://www.mdpi.com/article/10.3390/ani14182749/s1, Table S1: AIC of different models improved in the present work. Table S2: Outputs of the final model. Figure S1: Model evaluation with residuals.
