# Supplementary material for: Relating Wildlife Camera Trap Data to Tick Abundance: Testing the Relationship in Different Habitats
Source: Animals (Basel). 2024 Sep 23;14(18):2749. doi: 10.3390/ani14182749 (PMC11429502; doi:10.3390/ani14182749)
Supplement: Supplementary file 1 [file animals-14-02749-s001.zip › animals-3176745-supplementary.pdf]

Table S1 – AIC model selection (Val Maira and Capanne di Marcarolo)

SD + NDVI + Altitude + WildRuminants_TO + WildBoar_TO + Mesocarnivores_TO	204
SD + NDVI + Altitude + s(WildRuminants_TO) + s(WildBoar_TO) + s(sMesocarnivores1)	198
SD + NDVI + Altitude + s(WildRuminants_TO, Altitude, k=4) + s(WildBoar_TO, Altitude, k=4) + sMesocarnivores1*Altitude	202
SD + NDVI + Altitude + s(WildRuminants_TO, Altitude, by=as.factor(Site), k=4) + s(WildBoar_TO, Altitude, by=as.factor(Site), k=4) + sMesocarnivores1*as.factor(Site)	187

Table S2 – Model output

```
Family: Tweedie(p=1.512)
Link function: log

Formula:
Numero1 ~ SD1 + NDVI1 + Altitudine + s(sRoe1, Altitudine, by = as.factor(Site
),
    k = 4) + s(sBoar1, Altitudine, by = as.factor(Site), k = 4) +
    sMesocarnivores1

Parametric coefficients:
                   Estimate Std. Error t value Pr(>|t|)
(Intercept)       0.0000000  0.0000000     NaN      NaN
SD1              -0.0660683  0.3100705  -0.213   0.8322
NDVI1             0.1788460  0.2148964   0.832   0.4096
Altitudine       -0.0008294  0.0004866  -1.705   0.0951 .
sMesocarnivores1 -0.0245347  0.1954541  -0.126   0.9007
---
Signif. codes:  0 ‘***’ 0.001 ‘**’ 0.01 ‘*’ 0.05 ‘.’ 0.1 ‘ ’ 1

Approximate significance of smooth terms:
                                        edf Ref.df     F p-value
s(sRoe1,Altitudine):as.factor(Site)1  2.000  2.000 4.486  0.0167 *
s(sRoe1,Altitudine):as.factor(Site)2  2.923  2.992 4.438  0.0104 *
s(sBoar1,Altitudine):as.factor(Site)1 1.793  1.955 2.281  0.0852 .
s(sBoar1,Altitudine):as.factor(Site)2 1.000  1.000 6.364  0.0152 *
---
Signif. codes:  0 ‘***’ 0.001 ‘**’ 0.01 ‘*’ 0.05 ‘.’ 0.1 ‘ ’ 1

Rank: 14/15
R-sq.(adj) =  0.101   Deviance explained = 43.4%
-REML = 91.609  Scale est. = 1.6992    n = 57
```

Figure S1 – Gam model check

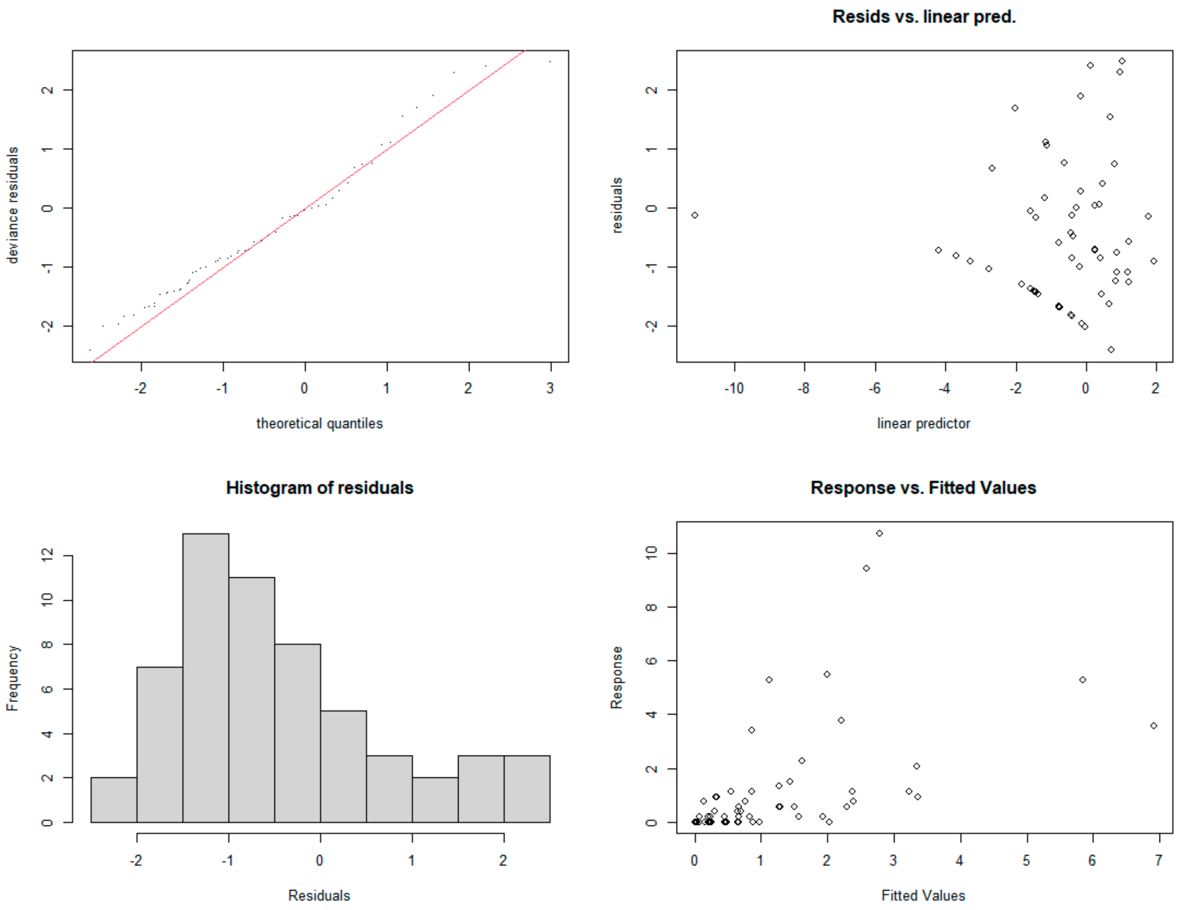
deviance residuals
theoretical quantiles
Resids vs. linear pred.
residuals
linear predictor
Histogram of residuals
Frequency
Residuals
Response vs. Fitted Values
Response
Fitted Values
